# Predictive model of hypoxemia after shoulder arthroscopy: A retrospective observational study

**DOI:** 10.1097/MD.0000000000032275

**Published:** 2022-12-09

**Authors:** Fei Lin, Xue Gong, Guangchun Lei, Xiong Wang, Cheng Chen, Lan Zhang

**Affiliations:** a Department of Anesthesia and Pain Medicine, Affiliated Hospital of Chengdu University, Chengdu, China; b Department of Anesthesia, The Seventh People’s Hospital of Chengdu, Chengdu, China; c Department of Anesthesia, Sichuan Provincial Orthopedic Hospital (Chengdu Sports Hospital and Chengdu Research Institute for Sports Injury), Chengdu, China.

**Keywords:** B-line, hypoxemia, post-anesthesia care unit, predictive model, shoulder arthroscopic surgery

## Abstract

The study is aimed to establish a predictive model of hypoxemia after shoulder arthroscopy. The predictive model was based on a retrospective study with 756 patients who underwent shoulder arthroscopic surgery in Sichuan Orthopaedic Hospital from June 2019 to December 2020. Independent risk factors of hypoxemia in the post-anesthesia care unit (PACU) were screened out by the binary logistics regression and the primary predictive model was completed, which was evaluated by the receiver operating characteristic (ROC) curve and Hosmer–Lemeshow goodness-of-fit test. A separate cohort of 324 patients in the PACU from January 2021 to June 2021 was enrolled to validate the predictive model. Seven hundred fifty-six patients and 19 variables were enrolled in the binary logistics regression and 324 patients were validated by the primary predictive model. Logistics regression showed that application of irrigating solution ≥20 L, age, body mass index, and number of B-lines were independent risk factors of hypoxemia in the PACU (*P* < .05). The risk predictive model of hypoxemia in the PACU was established according to those factors. The model was validated by the Hosmer–Lemeshow test and the area under the curve of ROC was 0.823. The model area under the curve of external effect subject ROC was 0.870. The risk predictive model established in our study can predict the risk of hypoxemia in the PACU well and have good efficacy.

## 1. Introduction

As a minimally invasive surgical procedure, shoulder arthroscopic surgery has become a routine method to diagnose and treat various shoulder joint diseases. Compared with open surgery, shoulder arthroscopy has obvious advantages such as less trauma, high accuracy, strong pertinence, quick postoperative recovery, and short hospital stay.^[1]^ Despite these benefits, it still has some disadvantages. For a better surgical view, it requires a large irrigation fluid to enlarge the joint cavity, which may lead a serious extravasation to the adjacent soft tissues. It may break the shoulder capsule, leading the irrigation to permeate into outer space. As a result, it may lead to the happening of significant edema of the face, neck, and chest tissues, tracheal compression, upper respiratory tract obstruction, and pulmonary edema. For swelling of the adjacent neck, chest, and facial tissues, most patients will be in lack of apparent syndrome within 2 days after surgery. However, in severe cases, abundant irrigation will flow into the pharyngolaryngeal and paratracheal space, which can lead to upper respiratory obstruction,^[2,3]^ hypoxemia, and even pulmonary edema.^[4]^ Complications caused by irrigation have been paid more and more alertness.^[5]^ Ultrasound is a reliable technique for monitoring various pulmonary edema. Compared with X-ray, it has the advantages of no radiation and realtime.^[6]^ The number of B-lines (NB) from ultrasound also plays an important role in evaluating the severity of pulmonary edema.^[7]^

For a better surgical view, shoulder arthroscopic surgery requires a lot of irrigation in some patients (>20 L). Unexplained hypoxemia can be found in patients, however, it is worth noting that those hypoxemia after shoulder arthroscopy can be life-threatening^[8,9]^ Early prevention of hypoxemia after shoulder arthroscopy is a highly concerning issue for anesthesiologists.^[10]^ Therefore, models can accurately predict the occurrence of hypoxemia, especially under the common action of some risk factors.^[11]^ In other words, the predictive model was used as a prediction tool to analyze related factors such as sex, age, body mass index (BMI), operation type, operation site, operation time, irrigation amount, B-line of lung, and so on. It can accurately predict the independent risk factors of hypoxemia after shoulder arthroscopy, which can provide a reliable basis for early clinical intervention.

This study aimed to explore the risk factors for hypoxemia after shoulder arthroscopy and to build a model to predict the probability of hypoxemia. Unlike previous studies, this study comprehensively analyzed the relationship between multiple independent risk factors and hypoxemia after shoulder arthroscopy. In addition, we used a separate cohort from January 2021 to June 2021 data to validate it.

## 2. Materials and Methods

### 2.1. Patients

A retrospective study was conducted on a primary cohort of patients who underwent shoulder arthroscopic surgery between June 2019 and June 2021 at the Sichuan Orthopaedic Hospital (Chengdu, China). Inclusion criteria included the following: patients who only underwent the shoulder arthroscopic surgery. Exclusion criteria were as follows: abnormal anatomical structures in the neck and chest, liver and kidney insufficiency, severe anemia, uncontrolled diabetes (fasting blood glucose ≥15 mmol/L), uncontrolled hypertension (systolic blood pressure [BP] ≥ 160 mm Hg, diastolic BP ≥ 100 mm Hg), arthroscopic conversion to open surgery, unclear lung tissue structure under ultrasound, preoperative arterial blood gas PaO_2_/FiO_2_ ≤300, the arterial blood gas analysis was not completed during the anesthesia recovery room or the oxygen concentration was not marked in the arterial blood gas analysis, and the study variable was missing.

From June 2019 to June 2021, an independent cohort of consecutive patients who underwent shoulder arthroscopic surgery was studied, using the same inclusion and exclusion criteria. These patients signed informed consent forms.

This study was approved by Sichuan Orthopaedic Hospital Research Ethics Committee (2019SGKL-004-01) and registered in China Clinical Trial Center (Registration number: 1900023793).

### 2.2. Methods

With the patient entering the operating room with a detailed history and a complete physical examination, the basic conditions of situation such as BP, pulse, pulse oxygen saturation and electrocardiogram were monitored. After intravenous channels were established, sodium lactate Ringer solution was injected. Then invasive BP was monitored by nonoperative radial artery puncture under local anesthesia. All patients received continuous interscalene brachial plexus block via posterior approach to provide complete intraoperative and postoperative analgesia (refer to Steven C Borene ^[12]^). To inject 20 mL 0.2% ropivacaine, the block effect was measured by acupuncture 20 minutes later.

Anesthesia with intravenous injection of atropine 0.01 mg/kg, sufentanil 0.2 to 0.3 μg/kg, propofol 2 to 4 mg/kg, and rocuronium 0.6 mg/kg, the patient will be conducted by endotracheal intubation and machine-controlled breathing with tidal volume 8 to 10 mL/kg, respiratory rate 10 to 12/min and the end of respiratory carbon dioxide 35 to 45 mm Hg. During the operation, propofol 2 to 4 mg kg^−1^ h^−1^ was injected intravenously, 1 to 2% sevoflurane was inhaled, and sufentanil was intermittent for 10 to 15 μg. Rocuronium 20 to 30 μg maintains anesthesia. Place a catheter after anesthesia and empty the residual urine volume before the operation. During the operation, the mean arterial pressure was maintained at 60 to 75 mm Hg. If the BP exceeded the expected range, adjust the depth of anesthesia, and give nitroglycerin or ephedrine if necessary. The patients were placed in the lateral position, and the joint cavity lavage was performed by gravity fluid control system, with 0.9% normal saline (3 L/bag) as the lavage fluid. At the end of the operation, neostigmine 0.2 mg/kg, an antagonist of muscle relaxant, was routinely given, and patients were delivered to the post-anesthesia care unit (PACU) after extubating.

The primary outcome was number counting of lung B-lines in before nerve block and after surgery. NB from ultrasound is an important diagnostic indicator of pulmonary edema, which were examined by Naviu color ultrasonic diagnostic system with low-frequency linear array probe (frequency 3.5–5.5 MHz), and 28 intercostal space scanning methods were used to measure NB of bilateral parasternal line, central clavicular line, anterior axillary line, central axillary line and posterior axillary line according to Picano Eugenio.^[6,7]^ The secondary outcomes included the following information of the patient: sex, age, BMI, American Society of Anesthesiologists grade, hypertension, preoperative and postoperative hemoglobin concentration, type of surgery, duration of surgery, irrigation amount, volume of fluid in and out at intraoperative, blood loss, urine volume, dosage of opioids and muscle relaxants and the incidence of postoperative hypoxemia. Postoperative hypoxemia was defined as the lowest PaO_2_/FiO_2_ in arterial blood gas ≤300 after oxygen inhalation through the nasal.^[13]^

### 2.3. Statistical analysis

All variables were presented as continuous variables or categorical variables. We excluded variables which were missing in >25% of cases. We used multiple interpolation to impute variables which were missing in <25% of cases. The original data set was divided into 2 sets randomly, including a training set (70% of data), and a validation set (30% of data). We used the training set to establish the predictive model. The validation set was utilized to estimate the accuracy of the model.

Statistical analysis was performed by SPSS 25.0 in Windows (SPSS, Chicago, IL) to identify risk factors. Measurement data in line with normal distribution were expressed as mean ± SD, and tested by *t* test. Measurement data which did not coincide with normal distribution were expressed as median (Q1, Q3), and tested by Wilcoxon Sign Rank Test. Categorical variables were grouped based on clinical findings, and decisions on the groups were made before modeling. The results were compared using the χ^2^ test or Fisher exact test. A value of *P* < .05 was considered statistically significant. *P* < .05 was considered statistically significant in logistic regression analysis. In terms of Hosmer–Lemeshow goodness of fit test, *P* < .05 was considered statistically significant, and the fitting degree was considered good. Receiver operating characteristic (ROC) curve was used to evaluate the model effect. The larger the area under the ROC curve (AUC) was, the better the model effect would be. The 324 consecutive patients admitted to PACU after shoulder arthroscopy from January 2021 to June 2021, were selected for external calibration of the model (the number of external calibration patients was >1/5 of the model group).

## 3. Results

### 3.1. Patient characteristics

All 756 patients were included in the trial without invalidation of anesthesia and changes in surgical methods. Demographic and surgical data were listed in Table 1.

**Table 1:** Patients of clinical data between the 2 groups (n = 756).

Variable	Hypoxemia group (n = 116)	Non-hypoxemia group (n = 640)
Age (yr)	56.8 ± 13.3	51.6 ± 14.4
Sex, n (%)		
Male	44 (37.9)	300 (46.9)
Female	72 (62.1)	340 (53.1)
Height (cm)	160.2 ± 8.1	161.5 ± 9.2
Weight (kg)	67.1 ± 11.2	62.7 ± 11.2
Body mass index (kg/m^2^)	26.1 ± 4.0	24.0 ± 3.6
ASA, n (%)		133/155/32
I	48 (41.4)	266 (41.6)
II	56 (48.3)	310 (48.4)
III–IV	12 (10.3)	64 (10)
Operation type, n (%)		
Dislocation of shoulder joint	18 (15.5)	92 (14.4)
Rotator cuff injury	88 (75.9)	490 (77.5)
Fracture of shoulder pelvis	10 (8.6)	58 (9.1)
Operation side, n (%)		
Left	23 (19.8)	166 (25.9)
Right	93 (80.2)	474 (74.1)
Preoperative MAP (mm Hg)	110.1 ± 14.3	109.6 ± 13.7
Operation time (min)	106.5 ± 35.6	111.2 ± 37.0
Anesthesia time (min)	169.1 ± 39.2	199.2 ± 40.5
Irrigating solution (L)	28.4 ± 11.2	23.6 ± 12.7
Average irrigation speed (mL/min)	162.1 ± 75.0	272.6 ± 79.6
Propofol (mg)	385.7 ± 94.6	417.7 ± 152.0
Sufentanil (μg)	23.8 ± 8.4	21.8 ± 8.7
Rocuronium (mg)	51.2 ± 9.6	53.8 ± 20.5
Sevoflurane (mL)	38.4 ± 7.8	40.7 ± 12.0
Ropivacaine (mg)	40	40
Venous intake (mL/kg)	7.1 ± 0.3	7.3 ± 0.7
Urine volume (mL)	212.1 ± 156.2	270.2 ± 224.7
Number of preoperative B-lines, n	0 (0, 2)	0 (0, 0)
Number of postoperative B-lines, n	5 (0, 7)	0 (0, 4)
Bleeding volume (mL)	27.9 ± 11.2	29.1 ± 30.5

ASA = American Society of Anesthesiologists, MAP = mean arterial pressure.

### 3.2. Predictive model for hypoxemia

The results of the univariate analysis are listed in Table 2. Multivariate analysis demonstrated that application of irrigating solution ≥20 L, age, BMI, and postoperative number of postoperative B-lines were independent risk factors of hypoxemia in PACU (*P* < .05). In another word, the odds ratio (OR) value for age was 1.065 (95% confidence interval [CI] 1.030–1.101), indicating a 0.101-fold increase in the probability of hypoxemia with each 1-year increase in age. The OR value of BMI was 1.125 (95% CI 1.041–1.215), indicating a 0.125-fold increase in the probability of hypoxemia for each 1-unit increase in BMI. The OR value of lavage ≥20 L was 5.505 (95% CI 2.314–13.095), indicating that the probability of hypoxemia in PACU of patients with irrigating solution ≥20 L was 5.505 times that of patients with irrigating solution <20 L. The OR value of NB after surgery was 1.084 (1.026–1.145), indicating that the probability of hypoxemia in PACU increased 0.084 times for each additional B line after surgery. (Table 2).

**Table 2:** Factors associated with hypoxemia after shoulder arthroscopic surgery.

Variable		Univariate analysis			Multivariate analysis		
		OR	95% CI	*P* value	OR	95% CI	*P* value
Sex	0 (female)	Reference					
	1 (male)	0.643	0.361–1.147	.135			
Age (yr)		1.080	1.046–1.116	<.001	1.065	1.030–1.101	<.001**
Body mass index (kg/m^2^)		1.152	1.074–1.236	<.001	1.125	1.041–1.215	.003**
ASA	I	Reference					
	II	1.001	0.554–1.810	.997			
	III–IV	1.039	0.392–2.753	.939			
The operation type	Dislocation of shoulder joint	Reference					
	Rotator cuff injury	1.878	0.710–4.962	.204			
	Shoulder glenoid rim fractures	2.800	0.807–9.715	.105			
The operation side	0 (Left)	Reference					
	1 (Right)	2.261	1.030–4.964	.042			
Hypertension	0 (None)	Reference					
	1 (All)	0.794	0.297–2.112	.645			
Postoperative hemoglobin concentration (g/dL)		0.996	0.982–1.011	.633			
Operation time (min)		0.996	0.988–1.004	.370			
Irrigating solution (L)	0 (<20)	Reference					
	1 (≥20)	7.756	3.416–17.607	<.001	5.505	2.314–13.095	<.001**
Propofol (mg)		0.998	0.996–1.000	.124			
Sufentanil (μg)		1.022	0.995–1.050	.110			
Rocuronium (mg)		0.991	0.947–1.009	.347			
Sevoflurane (mL)		0.981	0.995–1.008	.165			
Venous intake (mL)		0.999	0.997–1.000	.149			
Urine volume (mL)		3.457	0.997–1.000	.063			
Bleeding volume (mL)		0.975	0.950–1.002	.070			
Number of preoperative B-lines		1.467	1.169–1.843	.001			
Number of postoperative B-lines		1.104	1.042–1.169	.001	1.084	1.026–1.145	<.001**

ASA = American Society of Anesthesiologists, CI = confidence interval, OR = odds ratio.

*P < .05.

** *P* < .01.

According to the transformation of independent risk factors and logistics equation, and the method of Nashef,^[14]^ the risk prediction model of PACU hypoxemia after shoulder arthroscopy was obtained:

SCORE = 0.063 × age + 0.081 × number of postoperative B lines + 0.118 × BMI + 1.706 × lavage fluid ≥20 L


$$\begin{array}{l} P=\frac{e^{\left(-9.787+SCORE\right)}}{e^{\left(-9.787+SCORE\right)}+1} \end{array}$$

where *e* is the base of natural logarithm, *e* = 2.71828.

**Figure 1.**
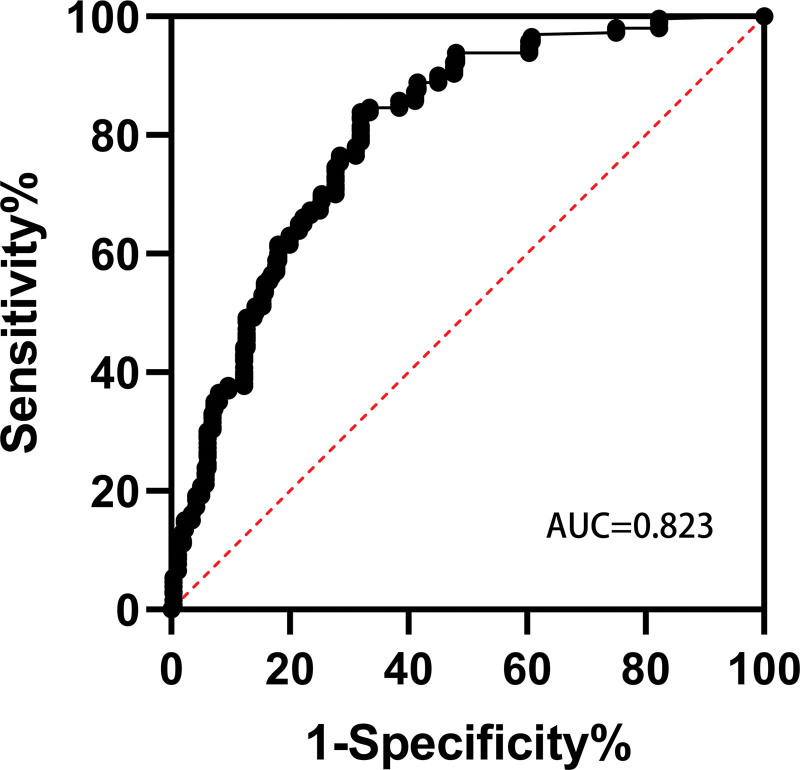
ROC for the prediction model AUC was 0.823 (95% confidence interval 0.771–0.875). AUC = area under the receiver operating characteristic curve, ROC = receiver operating characteristic curve.

Hosmer–Lemeshow goodness of fit test was used to evaluate the model, and the significance level was 0.05. The *P* value was .988 (*P* > .05), passing the check. Curve ROC was used to evaluate the accuracy of the model. The model AUC was 0.823 (Fig. 1), which passed the verification. These results indicate that the predictive model of hypoxemia in PACU after shoulder arthroscopy is effective.

**Figure 2.**
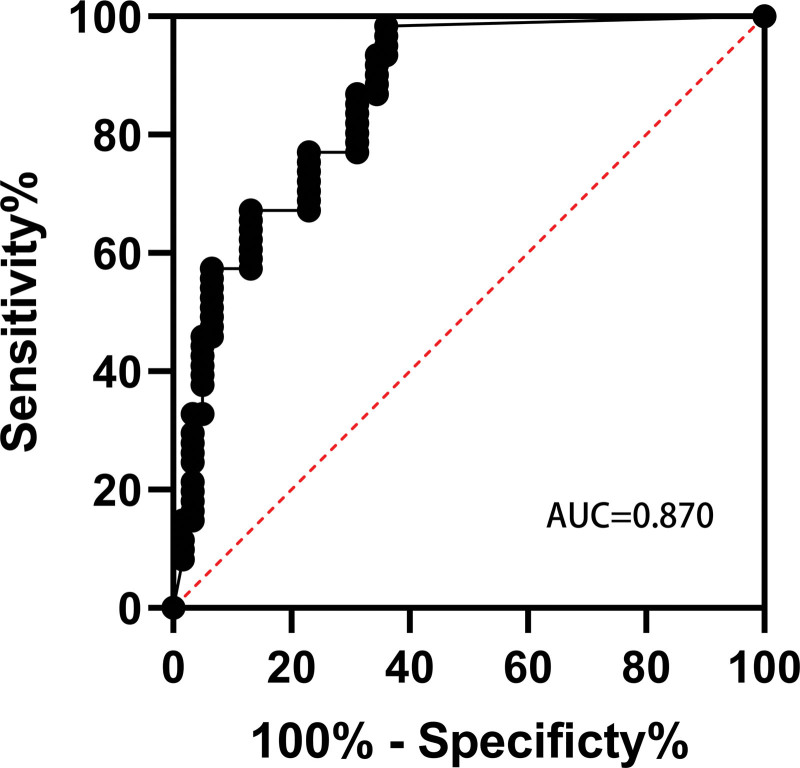
ROC for the external effect prediction model AUC was 0.870 (95% confidence interval 0.765–0.976). AUC = area under the receiver operating characteristic curve, ROC = receiver operating characteristic curve.

The model was externally validated in 324 consecutive patients admitted to the PACU after shoulder arthroscopy from January 2021 to June 2021. In this group, the lowest PaO2/ FiO2 value in PACU arterial blood gas was ≤300 in 11 patients, and the incidence of hypoxemia was 14%. In the evaluation results, the AUC = 0.870 was used to verify the effectiveness, indicating that the external effectiveness of the model was good (Fig. 2). Prediction accuracy = number of positive and negative cases of correctly prediction/total = (5 + 68)/79 = 92.41%; misclassification rate = number of positive and negative cases of false prediction/total = 5/79 = 7.59%; sensitivity = number of correctly predicted positive cases/number of actual positive cases = 5/11 = 45.45%; specificity = number of correctly predicted negative cases/number of actual negative cases = 68/68 = 100%; negative likelihood ratio = (1–sensitivity)/specificity = (1–40%)/100% = 0.6. Jordon coefficient is 0.546.

## 4. Discussion

In this study, there were 4 independent factors affecting the occurrence of hypoxemia in PACU after shoulder arthroscopy. The 4 independent influencing factors were lavage fluid ≥20 L, age, BMI, and postoperative B-line number of lung. The results of this study are consistent with those of Blumenthal,^[15]^ Memon M,^[9]^ Abutalib RA,^[16]^ etc.

There is no upper limit of lavage fluid consumption in shoulder arthroscopic surgery. According to our study, the amount of lavage fluid ≥20 L was easy to cause respiratory obstruction and hypoxemia after surgery, which was consistent with the study of Memon M.^[9]^ Memon M^[9]^ estimated that the lavage dosage was from 20L to 36L according to the statistics from previous case reports with symptoms, and speculated that it was safe to use lavage dosage <20L. Our study confirmed Memon’s inferences with extensive data and found that patients with lavage fluid volume of 20L or more had a 5 times higher incidence of hypoxemia than patients with lavage fluid volume of <20 L. The possible reasons for the hypoxemia caused by the extravasation of lavage fluid in shoulder arthroscopy are as follows: the lavage fluid retained in the tissues of the operation site, permeated to the neck and chest, and infiltrated into the paratracheal space and the para-carotid space through the cervical tissue space, resulting in tracheal compression and even respiratory tract obstruction^[8]^; respiratory auxiliary muscle groups and thoracic compliance may be affected by chest tissue edema, which reduces the radian of respiratory movement and causes hypoxemia; there may also be infiltration of lavage fluid in the pleural cavity or the interstitial lung,^[16]^ which limits the exchange function of the lung, causes hypoxia, and may even cause pleural effusion and pulmonary edema, which endangers the life safety of the patient. Gogia et al^[17]^ reported a case of hypoxemia after extubation and pulmonary edema after re-intubation. Therefore, early, timely, and accurate prediction of the occurrence of hypoxemia can prevent the occurrence of these adverse events early and effectively.

The study of Bartoszewski et al also showed that the prevalence of rotator cuff injury in elderly patients >60 years was >25%, and that in elderly patients >80 years was 62%.^[18]^ This study found that patients aged 65 and over accounted for 21% of shoulder arthroscopic surgery, and patients aged ≥50 accounted for 68%, indicating that the majority of middle-aged and elderly patients underwent shoulder arthroscopic surgery.^[18]^ This study showed that age was an independent risk factor for hypoxemia after shoulder arthroscopic surgery, and the risk of hypoxemia increases with age, so we need to pay more attention to the occurrence of adverse events of hypoxemia in middle-aged and elderly patients. With the increase of age, the subcutaneous tissue becomes relaxed, especially in patients aged ≥50, so older patients are more likely to have lavage extravasation, leading to hypoxemia, which is consistent with the study of Steven.^[12]^ Arthroscopic shoulder surgery may cause of the hypoxemia of the tissue edema, for which both sides of the tissue are connected by the skin, subcutaneous fat and bone, and as lavage fluid seepage path, the subcutaneous fat layer leads to lavage fluid extravasation into adjacent tissue edema was recorded, which also can lead to airway complications such as shift, airway obstruction and hypoxemia. Geraldes^[19]^ found that, with the increase of age, diseased small vessels caused decreased vascular reactivity, and relatively insufficient perfusion led to hypoxemia, so there was a significant correlation between age and postoperative hypoxemia. According to the study of Schouten,^[20]^ the balance of lung renin-angiotensin system decreases with age, leading to increased inflammation and lung injury. These studies were consistent with our study, indicating that patients with increasing age are at significantly increased risk of hypoxemia after shoulder arthroscopic surgery.

Kendale^[21]^ found that the incidence of perioperative hypoxemia in patients with normal BMI was 16%, that the perioperative incidence of hypoxemia was 28% in patients with a BMI >30 kg/m^2^ and 35% in patients with a BMI >40 kg/m^2^. This study also found that patients had an increasing risk of postoperative hypoxemia during shoulder arthroscopic surgery with BMI increasing, which was consistent with the results of Kendale^[21]^ and Steier.^[22]^ Steier^[22]^ found that the special physiological structure of obese patients resulted in decreasing lung and chest wall compliance, increasing airway resistance, and reducing functional residual capacity, leading to intrapulmonary shunt, alveolar collapse, and ventilate/perfusion mismatch. Therefore, the increase of BMI was closely related to the incidence, severity, and duration of perioperative hypoxemia. Therefore, patients with high BMI are at higher risk of hypoxemia after shoulder arthroscopy.

Memon M^[9]^ found that external joint leakage can lead to a variety of complications, including edema, related tracheal compression and airway obstruction, and even respiratory damage such as pleural effusion and pulmonary edema. Ultrasound has been widely used in emergency and intensive care units to assess pulmonary edema. On pulmonary ultrasound, NB is even more sensitive than X-rays in assessing the severity of pulmonary edema and can be detected at a subclinical stage before the clinical manifestations of pulmonary edema appear. Based on NB, we were able to assess whether the patient had severe pulmonary edema. The possible causes of pulmonary edema are as follows: during surgery, a large amount of fluid is spilt from the surrounding soft tissue, and part of the fluid is absorbed by the blood, leading to an increase in blood circulation; the patient’s chest and trachea compression can result in incomplete airway obstruction. When breathing is difficult, negative chest pressure increases, promoting venous return and increasing pulmonary artery pressure; the increase of thoracic hypoxia and negative pressure can decrease cardiac output, which further increase pulmonary vein and microvascular pressure. Therefore, increased pulmonary hydrostatic pressure can lead to fluid entering the pulmonary interstitial lung and increasing extravascular pulmonary fluid as pulmonary edema. NB can be assessed for the presence of pulmonary edema, which was an independent risk factor for the development of hypoxemia.

## 5. Limitations

This study had some limitations. The sample size was not large enough. It only focused on the type of shoulder arthroscopic surgery and cannot promote the actual occurrence of hypoxemia in clinical work of all surgical patients. However, shoulder arthroscopy is a type of surgery with high incidence of hypoxemia. In-depth study on the specific population of shoulder arthroscopy can provide reliable basis for predicting hypoxemia.

## 6. Conclusions

In conclusion, the risk prediction model of PACU hypoxemia after shoulder arthroscopy established in this study can better predict the risk of PACU hypoxemia after shoulder arthroscopy, and the prediction efficiency is good. Large irrigating solution, increased age, increased BMI, and increased postoperative B-line were independent risk factors for hypoxemia after shoulder arthroscopic surgery.

## Acknowledgements

The authors thank Wenfan Gan for the help of collecting follow-up data and Qihai Wan for valuable advice on data analysis.

## Author contributions

**Conceptualization:** Xue Gong, Lan Zhang.

**Data curation:** Xue Gong, Lan Zhang, Xiong Wang.

**Formal analysis:** Xue Gong, Lan Zhang, Xiong Wang.

**Funding acquisition:** Xue Gong.

**Investigation:** Xue Gong, Guangchun Lei.

**Methodology:** Lan Zhang, Guangchun Lei.

**Project administration:** Lan Zhang, Guangchun Lei.

**Resources:** Lan Zhang, Guangchun Lei.

**Software:** Xue Gong, Guangchun Lei.

**Visualization:** Lan Zhang.

**Writing – original draft:** Fei Lin.

**Writing – review & editing:** Xue Gong, Lan Zhang, Guangchun Lei, Xiong Wang, Cheng Chen.
